# Anthelmintic effects of Podang mango (*Mangifera* *indica*) fruit peel waste extract through *in vivo* application on Indonesian Etawa goat production and health

**DOI:** 10.14202/vetworld.2024.1291-1298

**Published:** 2024-06-14

**Authors:** Endry Nugroho Prasetyo, Efi Rokana, Zein Ahmad Baihaqi, Samudi Samudi

**Affiliations:** 1 Department of Biology, Faculty of Science and Data Analitics, Institut Teknologi Sepuluh Nopember (ITS), Surabaya, Indonesia; 2 Divisions of Animal Husbandry, Faculty of Agriculture, Universitas Islam Kadiri, Kediri, Indonesia; 3 Research Center for Animal Husbandry, National Research and Innovation Agency (BRIN), Bogor, Indonesia; 4 Division of Agrotechnology, Faculty of Agriculture, Universitas Islam Kadiri, Kediri, Indonesia

**Keywords:** anthelmintic, feed additive, gastrointestinal helminth infection, goat, *Haemonchus contortus*, *Mangifera indica* L

## Abstract

**Background and Aim:**

The continuous use of anthelmintic drugs has led to global issues of resistance. One breakthrough to address this problem is the utilization of bio-anthelmintics derived from active compounds in agro-industrial waste. This *in vivo* study investigated the effectiveness of Podang mango (*Mangifera indica* L.) fruit peel waste extract for anthelmintic purposes, using concentrations up to 5%.

**Materials and Methods:**

This study included 28 Etawa crossbred goats aged 17 months. Goats were randomly assigned to four groups: A negative control, an aqueous fruit peel extract (AFPE) group at 2.5%, another AFPE group at 5%, and a positive control receiving ivermectin. Goats chosen had egg per gram (EPG) counts surpassing 1000 before exposure to *Haemonchus contortus*. For 7 days within a 9-week study, AFPE from Podang mangoes was given. On the 7^th^ day, the positive control group was administered ivermectin. AFPE dosage relied on the average abomasum fluid per kilogram of animal weight. The feeding regimen consisted of concentrate and *Pennisetum purpureum* cv. Mott is customized for the nutritional needs of livestock. Data on feed consumption, digestibility, average daily gain, percentage reduction in fecal egg count, body condition score, and clinical parameters were collected throughout the study.

**Results:**

At higher treatment levels (AFPE), there was a greater reduction in both EPG and fecal egg counts. The expected and normal ranges were maintained for consumption and digestibility. While body weight increased, FAMACHA parameters showed a decrease. Compared to the negative control group, substantial disparities (p < 0.05) existed for hemoglobin, red blood cells, and hematocrit in both the positive control and the treatment groups. Blood urea nitrogen and creatinine, indicative of liver and kidney health, were within normal ranges.

**Conclusion:**

At a concentration of up to 5%, Podang mango waste extract (AFPE) can function as a substitute for traditional helminth medicines or bio-anthelmintics in goats, enhancing their production.

## Introduction

Due to the expanding human population, the need for animal protein has escalated [1]. Goats, an alternative in Indonesia, are gaining popularity for meeting the increasing demand for animal proteins [2]. According to Livestock and Animal Health Statistics [3], goat farming in East Java has markedly expanded (goat population of 19,400,000 by 2022) in Indonesia. In Indonesia’s community-based goat farming, health issues due to gastrointestinal nematode infections pose significant challenges. Studies by Baihaqi *et al*. [4, 5] highlighted *Haemonchus contortus* as the most common nematode infecting small ruminants in Indonesia. Parasite-related problems pose significant challenges in goat farming across Indonesian landscapes. Effective management of gastrointestinal nematode infections in goats is crucial for maintaining their health and productivity as significant contributors to animal protein production. The global ruminant sector grapples with a significant issue: Parasite offspring increasingly resist chemical drugs due to their misuse. Researchers seek plant-derived active compounds to address drug resistance [6]. To tackle resistance issues, researchers are exploring new anthelmintic options. Baihaqi *et al*. [7] revealed the untapped potential of *Paraserianthes falcataria* bark waste. According to SEM findings, *P. falcataria* bark waste harbors plant compounds essential for helminth death through damage to the helminth cuticle. Baihaqi *et al*. [8] demonstrated through an *in vitro* study that *Carica pubescens* seed waste damages *H. contortus* cuticle structure, thus eliminating it. This study found that *Carica* seeds did not impact ruminal fermentation while lowering methane gas production, supporting earlier findings by Baihaqi *et al*. [9].

Studies are essential to verify the anthelmintic properties of active plant compounds extracted from agro-industrial waste in living organisms [6]. Analyzing hematological and blood chemistry parameters is required for evaluating an individual’s health status [10]. According to Hajare *et al*. [11], helminth infections of the digestive tract result in approximately 200,000 ruminant deaths globally. Štrbac *et al*. [12] underline the importance of investigating alternative anthelmintics, especially those derived from natural plant sources.

The present study explored the effectiveness of aqueous fruit peel extract (AFPE) for anthelmintic purposes, using concentrations up to 5%. This study, by examining hematological and blood chemistry markers systematically, shed light on AFPE’s potential as a long-term, viable treatment for gastrointestinal helminth infections in ruminants. We seek alternative anthelmintic treatments in line with the global effort.

## Materials and Methods

### Ethical approval

The research protocol was approved by the Animal Care and Use Committee of the Universitas Brawijaya (reference number 144-KEP-UB-2023).

### Study period and location

The study was conducted from July 2023 to October 2023 at Al Baihaqi Farm in Kediri, East Java, Indonesia. The samples were tested at the Faculty of Veterinary Medicine, Universitas Gadjah Mada, Indonesia.

### Plant material and extraction

Podang mango (*Mangifera indica* L.) fruit peel was collected from small- and medium-sized enterprises in Banyakan Village, Kediri, East Java, Indonesia (CV. Sumber Mulyo). Experiments on water extracts and phytochemical (qualitative and quantitative) analyses were conducted using the method described by Dicko *et al*. [13] with some modifications (the filtration was done four times using absorbent cotton).

### Experimental design

Twenty-eight 17-month-old Etawa crossbred male goats were randomly distributed among four treatment groups in this research. The goats weighed between 24 and 27 kg. For 7 days, goats infected with *H. contortus* from a local farming area (with egg per gram [EPG] count exceeding 1000) received consecutive AFPE treatments at doses of 2.5% and 5%. On the 7^th^ day, the positive control group was given a singular oral dose of ivermectin. Goats were housed individually with a diet of copra cake, palm oil cake, corn gluten feed, coffee peel, corn waste, cassava waste, molasses, premix, multivitamins, herbs, and Napier grass. An unlimited supply of water was available.

### Parasitological examination

The parasitological examination in this study involved the collection of fecal samples from the rectum of goats using the grab sampling method at various time points during the pre-study period and on days 0, 7, 14, 21, and 28. The McMaster method determined EPG as indicated by Baihaqi *et al*. [4]. Two gram of stool was transferred to a beaker and 58 mL of saturated sugar solution was added to reach a final volume of 60 mL. The tea strainer was used to stir and filter the mixture. A McMaster counting chamber received the fecal suspension extracted with a pipette. After a 2–3 min soak, the samples were observed under a microscope (VHX-7000 KEYENCE, Indonesia) (magnification 40×). The total EPG was determined by identifying and counting *H. contortus* nematode eggs.

### Body weight

Livestock were weighed once a week in the morning before their weekend feeding. Assessing body condition score (BCS) involves examining and feeling eight distinct body regions: The spinous and transverse processes, flanks, tuber coxae, and ischiatic tubers. The BCS assessment diagram employs a 1–4 scale (1 – very thin, 2 – skinny, 3 – moderate, and 4 – fat) [14].

### Feed consumption and digestibility

The dry matter (DM), organic matter (OM), and crude protein (CP) content of both concentrate and forage animal feed samples were analyzed for feeding consumption calculation [2]. Stool samples were collected within 24-h post-treatment with *M. indica* fruit peel extract. About 10% of the samples were first dried in the sun and then oven-dried at 60°C. A Wiley Mill grinding maching (https://www.thomassci.com/Equipment/Mills/_/Wiley-Mill-4-12-Horse-Power-Unit-Accessories) with a 1-mm sieve size was used to grind dry feces for seven consecutive days. The contents of DM, OM, and CP in composite fecal samples were determined as described by Lokapirnasari *et al*. [15].

### Blood samples and FAMACHA^©^ system

Three-milliliter syringes were used to draw blood from the jugular veins of goats. Blood samples were collected on day 0 and day 28. A blood test was carried out to determine hemoglobin (Hb) levels, erythrocyte count, packed cell volume (PCV), leukocyte count, leukocyte differential, and blood chemistry [16]. Using FAMACHA^©^ (University of Rhode Island, United States), the eyes were opened to assess animal health. The eyes’ upper and lower eyelids exhibited mucosal alterations. The color of the eyelids was compared to that of FAMACHA^©^ for assessing anemia caused by *H. contortus* infection [14]. The image’s color was used to assess anemia’s severity. Anemic degree was compared with PCV results subsequently.

### Statistical analysis

One-way analysis of variance was used to analyze data obtained from *in vivo* experiments. Duncan’s multiple-range test determined the statistical differences between the means. p < 0.05 was considered statistically significant. The data were analyzed using Statistical Package for the Social Sciences, version 20.0 (IBM Corp., NY, USA).

## Results and Discussion

### Qualitative and quantitative phytochemical analyses of Podang mangoes

Tables-1 and 2 present the findings of qualitative and quantitative phytochemical analyses. The analysis of active compounds such as tannins, alkaloids, flavonoids, saponins, and steroids from Podang APFE mangoes yielded positive qualitative findings, while the quantitative analysis revealed a total phenolic content of 9.8 (mgGallic acid equivalen/g dw), a flavonoid content of 6.3 (mg Rutin equivalent/g dw), and a total tannin content of 7.3%. The industrial waste is potentially valuable for multiple uses in the ruminant industry. The *in vitro* research proposes it as a potential alternative to control gastrointestinal helminth infections, decrease methane emissions, and safeguard proteins [5, 7, 8].

**Table-1:** Qualitative phytochemical analysis.

Phytochemicals	Tannin	Flavonoid	Saponin	Alkaloid	Steroid
AFPE samples	+	+	+	+	+

(+) Presence of constituents, (−) absence of constituents, AFPE=Aqueous fruit peel extract

**Table-2:** Quantitative phytochemical analysis of Podang mango.

Material	AFPE of Podang mango (%)
Tannin total (%)	7.3
Condensed tannin, CT (%)	4.9
Hydrolyzed tannin (%)	3.5
Total phenolic (mg GAE/g dw)	9.8
Flavonoids content (mg RE/g dw)	6.3

AFPE=Aqueous fruit peel extract of Podang mango, CT=Computed tomography, GAE=Gallic acid equivalent, RE=Rutin equivalent

### EPG-fecal egg count reduction (FECR) parameters

The FECR and EPG indices decreased in all groups, with the values presented in Table-3 for the negative control, 2.5% AFPE of Podang mango, 5% AFPE treatment, and positive control. During the study, improved nutrition led to a decrease in helminth egg count for the negative control group. About 100% of reduction in EPG was observed in the positive control group given ivermectin. About 2.5% and 5% of AFPE significantly reduced *H. contortus* egg production in thin-tailed goats. Hazarika *et al*. [17] observed that the extracts of *Nyctanthes arbor-tristis* and *Butea monosperma*, rich in tannins, saponins, flavonoids, and phenols, decreased FECR significantly. Mesquita-Sousa *et al*. [18] noted that *Citrus aurantium* var. is effective when used in combination. Dulcis exhibited improved FECR due to the presence of active compounds.

**Table-3:** Effect of AFPE Podang mango on EPG-FECR parameters.

Treatment group	EPG		FECR (%)

	Pre-treatment	Post-treatment	
Negative control	1385.29 ± 12.22^a^	1172.51 ± 26.39^b^	15.37
AFPE 2.5%	1283.28 ± 18.35^a^	673.31 ± 2.33^b^	47.54
AFPE 5%	1316.37 ± 24.52^a^	218.42 ± 3.11^b^	83.43
Positive control	1251.22 ± 16.21^a^	0.00 ± 0.00^b^	100.00

^a,b^Different superscripts on the same line indicate significant differences (p < 0.05). AFPE: Aqueous fruit peel extract of Podang mango, EPG-FECR=Egg per gram-fecal egg count reduction

### Nutrient intakes

Table-4 shows that the average consumption of DM, OM, and CP in all treatment groups with AFPE, regardless of the concentration (up to 5%), did not significantly differ between pre- and post-treatment stages (p > 0.05). The consumption of DM, OM, and CP by Arabi goats remains unaffected by *Albizia lebbeck* tannins, according to Ardeshiri *et al*. [19]. Faryabi *et al*. [20] reported no significant difference in feed consumption, water intake, final body weight, daily weight gain, or feed conversion ratio on substituting sheep feed with *Artemisia sieberi* leaves.

**Table-4:** Effect of AFPE Podang mango on DMI, OMI, and CPI (grams per day).

Treatment group	Parameter	Pre- treatment (g)	Post- treatment (g)
Negative control	DMI	869.16 ± 0.28	873.28 ± 0.17
	OMI	832.72 ± 0.29	839.62 ± 0.21
	CPI	193.71 ± 0.21	197.26 ± 0.16
AFPE 2.5%	DMI	851.73 ± 0.24	874.27 ± 0.14
	OMI	836.28 ± 0.37	840.53 ± 0.26
	CPI	185.73 ± 0.28	189.16 ± 0.25
AFPE 5%	DMI	821.73 ± 0.25	824.39 ± 0.16
	OMI	817.53 ± 0.16	821.27 ± 0.15
	CPI	171.88 ± 0.24	174.38 ± 0.25
Positive control	DMI	836.53 ± 0.29	841.39 ± 0.17
	OMI	827.36 ± 0.25	830.62 ± 0.16
	CPI	173.35 ± 0.89	177.32 ± 0.25

^a,b^Different superscripts on the same line indicate significant differences (p < 0.05). AFPE=Aqueous fruit peel extract of podang mango, DMI=Dry matter intake, OMI=Organic matter intake, CPI=Crude protein intake

### Nutrient digestibility

Digestibility parameters of DM, OM, and CP for goats in groups with up to 5% AFPE and the negative and positive control groups remained unchanged (p > 0.05) from pre- to post-treatment (Table-5). According to Ibidhi and Salim [21], goats given *Trigonella foenum graecum* L. seeds, known for phenolic compounds, experienced no substantial decrease in digestibility. Shilwant *et al*. [22] discovered varied outcomes: Their composite plant extract improved digestibility of DM and OM, while CP remained unaffected.

**Table-5:** Effect of AFPE Podang mango on nutrient digestibility (%).

Treatment group	Parameters	Pre- treatment (%)	Post- treatment (%)
Negative control	DM digestibility	72.19 ± 0.16	71.28 ± 0.24
	OM digestibility	73.31 ± 0.17	72.23 ± 0.25
	CP digestibility	74.11 ± 0.28	73.03 ± 0.28
AFPE 2.5%	DM digestibility	73.20 ± 0.21	72.17 ± 0.41
	OM digestibility	72.12 ± 0.17	71.16 ± 0.28
	CP digestibility	73.15 ± 0.24	72.04 ± 0.25
AFPE 5.0%	DM digestibility	73.06 ± 0.12	72.66 ± 0.15
	OM digestibility	72.13 ± 0.41	71.58 ± 0.34
	CP digestibility	73.25 ± 0.18	72.42 ± 0.21
Positive control	DM digestibility	72.36 ± 0.11	71.26 ± 0.25
	OM digestibility	72.24 ± 0.16	71.94 ± 0.23
	CP digestibility	73.12 ± 0.24	72.43 ± 0.21

^a,b^Different superscripts on the same line indicate significant differences (p < 0.05). AFPE=Aqueous fruit peel extract of Podang mango

### Effect of AFPE Podang mango on hematology parameters

In Table-6, Hb, red blood cells (RBC), and hematocrit levels significantly differed between groups treated with 2.5% and 5% of AFPE, as well as the positive control, versus the negative control group, before and after treatment (p < 0.05). No significant change in these parameters was observed for the untreated group with gastrointestinal nematodes (GIN) (negative control). Suppressing *H. contortus*’ EPG count was demonstrated by this. Dubey *et al*. [23] found goats treated with *Tecoma stans* seed ethanol extract had significant increases (p > 0.05) in white blood cells, Hb, RBC, and PCV compared to the untreated group. The increase in hematological variables in this study can be explained by the body’s reaction to *H. contortus*, a blood-feeding parasite causing gastrointestinal inflammation. According to Sambodo *et al*. [14], the negative control group had lower RBC and leukocyte parameters, indicating that GIN infection in goats could lead to anemia through blood loss.

**Table-6:** Effect of AFPE Podang mango on hematology parameters.

Treatment group	Hematology parameters	Pre-treatment	Post-treatment
Negative control	Hb (g/dL)	8.32 ± 0.17	8.94 ± 0.23
	HCT (%)	23.16 ± 0.44	23.95 ± 0.35
	MCV (fL)	40.19 ± 0.22	40.27 ± 0.15
	MCH (pg)	11.27 ± 0.25	12.28 ± 0.17
	MCHC (%)	33.24 ± 0.28	33.21 ± 0.18
	RBC (×10^6^/μL)	8.16 ± 0.89	8.46 ± 0.24
	Leukocyte (×10^3^/mm^3^)	16.25 ± 0.27	15.94 ± 0.25
	Neutrophil (%)	40.15 ± 0.29	39.22 ± 0.25
	Eosinophil (%)	29.58 ± 0.21	28.24 ± 0.44
	Basophil (%)	0.00 ± 0.00	0.00 ± 0.00
	Lymphocytes (%)	59.28 ± 0.21	57.24 ± 0.29
	Monocytes (%)	3.00 ± 0.00	3.00 ± 0.00
AFPE 2.5%	Hb (g/dL)	11.95 ± 0.21	13.57 ± 0.45
	HCT (%)	26.13 ± 0.25^b^	28.13 ± 0.18^a^
	MCV (fL)	40.14 ± 0.17^a^	37.12 ± 0.16^b^
	MCH (pg)	12.03 ± 0.13	11.36 ± 0.25
	MCHC (%)	27.51 ± 0.29	26.24 ± 0.21
	RBC (×10^6^/μL)	9.03 ± 0.15	11.14 ± 0.23
	Leukocyte (×10^3^/mm^3^)	14.03 ± 0.22^a^	11.27 ± 0.56^b^
	Neutrophil (%)	25.12 ± 0.28^a^	29.13 ± 0.15^b^
	Eosinophil (%)	32.04 ± 0.25^a^	26.22 ± 0.13^b^
	Basophil (%)	0.00 ± 0.00	0.00 ± 0.00
	Lymphocytes (%)	58.23 ± 0.14^a^	45.25 ± 0.21^b^
	Monocytes (%)	3.00 ± 0.00	3.00 ± 0.00
AFPE 5%	Hb (g/dL)	10.81 ± 0.18	13.24 ± 0.18
	HCT (%)	24.15 ± 0.12^b^	28.27 ± 0.15^a^
	MCV (fL)	39.13 ± 0.17^a^	35.12 ± 0.15^b^
	MCH (pg)	13.23 ± 0.15	11.53 ± 0.23
	MCHC (%)	27.14 ± 0.13	26.11 ± 0.24
	RBC (×10^6^/μL)	9.05 ± 0.21	11.88 ± 0.24
	Leukocyte (×10^3^/mm^3^)	14.28 ± 0.13^a^	11.05 ± 0.22^b^
	Neutrophil (%)	25.47 ± 0.21^a^	30.46 ± 0.12^b^
	Eosinophil (%)	28.33 ± 0.25^a^	23.86 ± 0.22^b^
	Basophil (%)	0.00 ± 0.00	0.00 ± 0.00
	Lymphocytes (%)	48.25 ± 0.25^a^	42.98 ± 0.21^b^
	Monocytes (%)	3.00 ± 0.00	3.00 ± 0.00
Positive control	Hb (g/dL)	10.35 ± 0.27	12.14 ± 0.21
	HCT (%)	25.26 ± 0.13^b^	27.83 ± 0.23^a^
	MCV (fL)	41.28 ± 0.24^a^	36.38 ± 0.21^b^
	MCH (pg)	12.88 ± 0.13	11.13 ± 0.31
	MCHC (%)	28.63 ± 0.28	27.12 ± 0.17
	RBC (×10^6^/μL)	8.18 ± 0.25	10.26 ± 0.25
	Leukocyte (×10^3^/mm^3^)	15.21 ± 0.28^a^	12.14 ± 0.27^b^
	Neutrophil (%)	27.35 ± 0.24^a^	31.22 ± 0.17^b^
	Eosinophil (%)	30.18 ± 0.42^a^	23.19 ± 0.27^b^
	Basophil (%)	0.00 ± 0.00	0.00 ± 0.00
	Lymphocytes (%)	50.02 ± 0.31^a^	43.02 ± 0.35^b^
	Monocytes (%)	3.00 ± 0.00	3.00 ± 0.00

^a,b^Different superscripts on the same line indicate significant differences (p < 0.05). AFPE=Aqueous fruit peel extract of Podang mango, Hb=Hemoglobin, RBC=Red blood cells, HCT=Hematocrit, MCH=Mean corpuscular hemoglobin, MCV=Mean corpuscular volume

### Effect of AFPE Podang mango on chemical blood parameters

Table-7 details the blood chemistry values of the goats before and after treatment. In the negative control groups, the aspartate transaminase (AST) and alanine transaminase (ALT) levels in goat blood remained unchanged, whereas a significant reduction (p < 0.05) was observed in the treated groups. A gastrointestinal helminth infection may cause damage to the digestive tract and liver, as indicated by this pattern. Administering extracts with active compounds led to decreased AST and ALT values in goats by Tanwar and Misra [24], suggesting an improvement in their liver tissue. Radostits *et al*. [25] reported an increased blood albumin level in the group given plant extracts and active compounds. The goats’ enhanced protein nutrition and improved liver function are suggested by this increase. In goats, decreased creatinine levels due to active plant compounds suggest a possible enhancement of kidney function or protein nutrition. Triterpenoids and saponins have been reported to exhibit hepatoprotective properties [26].

**Table-7:** Effect of AFPE Podang mango on blood chemical parameters.

Treatment group	Blood chemical parameters	Pre-treatment	Post-treatment
Negative control	TP (g/dL)	6.27 ± 0.14	5.98 ± 0.17
	Albumin (g/dL)	2.58 ± 0.11	2.49 ± 0.12
	Globulin (g/dL)	2.91 ± 0.28	2.83 ± 0.15
	Glucose (mg/dL)	45.58 ± 0.21	45.43 ± 0.18
	BUN (mg/dL)	15.82 ± 0.13	15.75 ± 0.16
	AST (IU/L)	130.26 ± 0.21	130.18 ± 0.13
	ALT (IU/L)	38.26 ± 0.14	38.37 ± 0.12
	Creatinine (mg/dL)	1.13 ± 0.27	1.12 ± 0.14
AFPE 2.5%	TP (g/dL)	5.84 ± 0.13	5.62 ± 0.29
	Albumin (g/dL)	3.58 ± 0.27	3.43 ± 0.18
	Globulin (g/dL)	2.92 ± 0.26	2.83 ± 0.21
	Glucose (mg/dL)	45.84 ± 0.17	45.62 ± 0.16
	BUN (mg/dL)	14.74 ± 0.21^a^	19.91 ± 0.27^b^
	AST (IU/L)	132.25 ± 0.16^a^	92.03 ± 0.24^b^
	ALT (IU/L)	37.01 ± 0.18^a^	25.22 ± 0.73^b^
	Creatinine (mg/dL)	1.22 ± 0.12	1.23 ± 0.15
	TP (g/dL)	5.84 ± 0.13	5.62 ± 0.29
	Albumin (g/dL)	3.58 ± 0.27	3.43 ± 0.18
AFPE 5%	TP (g/dL)	5.83 ± 0.12	5.63 ± 0.22
	Albumin (g/dL)	3.79 ± 0.17	3.64 ± 0.13
	Globulin (g/dL)	2.84 ± 0.26	2.75 ± 0.13
	Glucose (mg/dL)	45.32 ± 0.19	46.74 ± 0.14
	BUN (mg/dL)	16.22 ± 0.12^a^	22.27 ± 0.28^a^
	AST (IU/L)	142.17 ± 0.27^a^	93.92 ± 0.21^b^
	ALT (IU/L)	38.37 ± 0.13^a^	26.35 ± 0.23^b^
	Creatinine (mg/dL)	1.24 ± 0.13	1.25 ± 0.24
Positive control	TP (g/dL)	5.53 ± 0.21	5.41 ± 0.87
	Albumin (g/dL)	3.83 ± 0.27	3.72 ± 0.25
	Globulin (g/dL)	2.82 ± 0.16	2.79 ± 0.19
	Glucose (mg/dL)	57.13 ± 0.24	56.28 ± 0.18
	BUN (mg/dL)	15.24 ± 0.17^b^	21.23 ± 0.13^a^
	AST (IU/L)	127.22 ± 0.12^a^	98.21 ± 0.15^b^
	ALT (IU/L)	39.42 ± 0.15^a^	26.28 ± 0.13^b^
	Creatinine (mg/dL)	1.25 ± 0.23	1.24 ± 0.21

^a,b^Different superscripts on the same line indicate significant differences (p < 0.05). AFPE=Aqueous fruit peel extract of Podang mango, TP=Total protein, BUN=Blood urea nitrogen, AST=Aspartate transaminase, ALT=Alanine transaminase

The goats treated in the study had blood urea nitrogen (BUN) and creatinine levels within the normal range for average goats (10–35 mg/dL for BUN and 1.2–1.9 mg/dL for creatinine). Rams given Kyasuwa grass had a BUN value of 14 mg/dL. In desert Bighorn goats, the BUN reference interval is 5–28 mg/dL and the creatinine range is 1.6–2.6 mg/dL. 5 mg/mL AFPE Podang mango and ivermectin did not impact kidney function. At this concentration, AFPE Podang mangoes did not harm the kidneys. The study by Meenakshisundaram *et al*. [27] supports the observation that the given plant extracts did not impair kidney function. The plant extracts administration did not harm the kidneys based on normal BUN and creatinine levels.

### Effect of AFPE Podang mango on FAMACHA parameters

In Table-8, the statistical analysis showed significant effects on FAMACHA parameters for the positive control group and the 5% AFPE treatment group (p < 0.05), while no significant differences were observed between the negative control and the 2.5% AFPE group. Kumar *et al*. [28] emphasized the value of FAMACHA for detecting *Haemonchus* spp. infection and proposed *Carica papaya* seed extract as an effective alternative to control haemonchosis in goats. Sambodo *et al*. [14] observed an increase in FAMACHA values coinciding with hematological changes following AFPE administration.

**Table-8:** Effect of AFPE Podang mango on FAMACHA parameters.

Treatment group	Pre-treatment	Post-treatment
Negative control	4.16 ± 0.29	3.75 ± 0.26
AFPE 2.5%	3.99 ± 0.17	3.64 ± 0.18
AFPE 5.0%	3.95 ± 0.14^a^	2.28 ± 0.19^b^
Positive control	4.04 ± 0.18^a^	2.15 ± 0.31^b^

^a,b^Different superscripts on the same line indicate significant differences (p < 0.05). AFPE=Aqueous fruit peel extract of Podang mango

### Effect of AFPE Podang mango on body weight

In Table-9, the *in vivo* study results showed that goats in both the AFPE treatment and positive control groups had significantly higher average body weights (p < 0.05), whereas goats in the negative control group had lower average body weights. Jamarun *et al*. [29] reported a 65.25 g/head/day increase in body weight gain when supplementing *Rhizophora apiculata* leaves, Hay, and Fermented *Tithonia diversifolia* with active compounds. Morais-Costa *et al*. [30] found significant weight gain using *Piptadenia* *viridiflora* water extract (p < 0.05). 5% AFPE administration, along with positive controls, led to an increase in body weight likely from decreased helminth infections. The goat’s increase in body weight did not negatively impact feed consumption, digestibility, or health conditions, as indicated by consistent hematology and blood chemistry readings.

**Table-9:** Effect of AFPE Podang mango on body weight parameters.

Treatment group	Pre-treatment (kg)	Post-treatment (kg)
Negative control	25.41 ± 0.19^a^	23.19 ± 0.13^b^
AFPE 2.5%	26.84 ± 0.23^b^	31.27 ± 0.25^a^
AFPE 5.0%	27.93 ± 0.15^b^	32.63 ± 0.25^a^
Positive control	24.93 ± 0.24^b^	31.72 ± 0.27^a^

^a,b^Different superscripts on the same line indicate significant differences (p < 0.05). AFPE=Aqueous fruit peel extract of Podang mango

### Effect of AFPE Podang mango on BCS parameters

The BCS rose significantly (p > 0.05) by up to 5% in the AFPE treatment group and 5% in the positive control group. A decrease in helminth infections in goats corresponded to an improvement in their BCS (Table-10), implying that AFPE mango pods did not negatively impact their health and nutritional status. Mahachi *et al*. [31] showed that incorporating up to 25% *Sericea lespedeza* in goat feedlots suppressed *H. contortus* infections, maintaining body weight and BCS unchanged. Likewise, Soto-Barrientos *et al*. [32] found a close relationship between goats’ low BCS and severe gastrointestinal nematode infection.

**Table-10:** Effect of AFPE Podang mango on body condition score (BCS) parameters.

Treatment group	Pre-treatment	Post-treatment
Negative control	3.00 ± 0.27	2.85 ± 0.14
AFPE 2.5%	3.00 ± 0.14	3.32 ± 0.18
AFPE 5%	3.20 ± 0.16	3.58 ± 0.42
Positive control	3.00 ± 0.18	3.42 ± 0.25

^a,b^Different superscripts on the same line indicate significant differences (p < 0.05). AFPE=Aqueous fruit peel extract of Podang mango, BCS scale: 1=very thin, 2=skinny, 3=moderate, 4=fat

## Conclusion

About 5% of Podang mango peel waste usage shows potential as a bioanthelmintic without negatively impacting livestock productivity and health. The study effectively decreased EPG while keeping feed consumption and digestibility constant, boosted body weight, reduced FAMACHA scores, and kept BUN and creatinine levels within normal limits.

## Authors’ Contributions

ENP, ZAB, ER, and SS: Designed the study, collected the samples, and performed the examinations. ZAB: Conducted field surveys. All authors have drafted, revised, and approved the final manuscript.
